# Comprehensive molecular profiling of advanced/metastatic olfactory neuroblastomas

**DOI:** 10.1371/journal.pone.0191244

**Published:** 2018-01-11

**Authors:** Jasmina Topcagic, Rebecca Feldman, Anatole Ghazalpour, Jeffrey Swensen, Zoran Gatalica, Semir Vranic

**Affiliations:** 1 Association of Basic Medical Sciences of Federation of Bosnia and Herzegovina, Sarajevo, Bosnia and Herzegovina; 2 Caris Life Sciences, Phoenix, Arizona, United States of America; 3 Department of Pathology, Clinical Center and School of Medicine, University of Sarajevo, Sarajevo, Bosnia and Herzegovina; 4 College of Medicine, Qatar University, Doha, Qatar; University of Michigan, UNITED STATES

## Abstract

Olfactory neuroblastoma (ONB) is a rare, locally aggressive, malignant neoplasm originating in the olfactory epithelium in the nasal vault. The recurrence rate of ONB remains high and there are no specific treatment guidelines for recurrent/metastatic ONBs. This study retrospectively evaluated 23 ONB samples profiled at Caris Life Sciences (Phoenix, Arizona) using DNA sequencing (Sanger/NGS [Illumina], n = 15) and gene fusions (Archer FusionPlex, n = 6), whole genome RNA microarray (HumanHT-12 v4 beadChip, Illumina, n = 4), gene copy number assays (chromogenic and fluorescent *in situ* hybridization), and immunohistochemistry. Mutations were detected in 63% ONBs including *TP53*, *CTNNB1*, *EGFR*, *APC*, *cKIT*, *cMET*, *PDGFRA*, *CDH1*, *FH*, and *SMAD4* genes. Twenty-one genes were over-expressed and 19 genes under-expressed by microarray assay. Some of the upregulated genes included *CD24*, *SCG2*, and *IGFBP-2*. None of the cases harbored copy number variations of *EGFR*, *HER2* and *cMET* genes, and no gene fusions were identified. Multiple protein biomarkers of potential response or resistance to classic chemotherapy drugs were identified, such as low ERCC1 [cisplatin sensitivity in 10/12], high TOPO1 [irinotecan sensitivity in 12/19], high TUBB3 [vincristine resistance in 13/14], and high MRP1 [multidrug resistance in 6/6 cases]. None of the cases (0/10) were positive for PD-L1 in tumor cells. Overexpression of pNTRK was observed in 67% (4/6) of the cases without underlying genetic alterations. Molecular alterations detected in our study (e.g., Wnt and cKIT/PDGFRA pathways) are potentially treatable using novel therapeutic approaches. Identified protein biomarkers of response or resistance to classic chemotherapy could be useful in optimizing existing chemotherapy treatment(s) in ONBs.

## Introduction

Olfactory neuroblastoma (ONB), also called esthesioneuroblastoma, is a rare, locally aggressive, malignant neoplasm originating in the specialized sensory neuroepithelial olfactory cells found in the upper part of the nasal cavity [1]. Multiple modalities are currently in use to treat ONB, including surgical resection, radiotherapy, and chemotherapy. Numerous studies confirmed that a combination of surgery and radiotherapy is the treatment of choice for the majority of primary-site ONBs [2–6]. Advanced and metastatic ONBs are usually treated with classic chemotherapy, including etoposide, ifosfamide, cisplatin, cyclophosphamide, vincristine, doxorubicin, and nitrogen mustard [3,7]. However, due to the unpredictable biological behavior of the tumor and a lack of consensus on traditional treatment modalities [2,3], the recurrence rate of ONB remains high and effective treatment guidelines for high-grade ONBs are yet to be developed.

In recent years, with the advancement of molecular diagnostic methods, the focus has been on developing individualized targeted therapies for treating different types of cancer [8]. Cytogenetic studies on ONB revealed diverse and complex genomic imbalances in entire chromosomes and chromosome segments [9–11]. Several studies have reported copy number changes in ONB including gains at 7q11 and 20q and deletions at 2q, 5q, 6p, 6q, and 18q [10], as well as novel chromosome aberrations that have not been previously described. In addition, some chromosome regions could be implicated in the tumor progression and metastases formation [1,12]. These results not only indicate complex molecular processes underlying ONB, but also point to the need for a more detailed molecular characterization of ONBs at different stages of tumor progression.

Currently only a few studies have investigated genomic landscape of ONBs, using different sequencing techniques [13–17]. Furthermore, in three of these studies the potential of targeted therapy with specific drugs was explored. Weiss et al. [13] performed whole genome sequencing (WGS) on paired normal and tumor DNA from a patient with metastatic ONB. They detected mutations specific only to the metastatic ONB sample (i.e., in *KDR*, *MYC*, *SIN3B*, and *NLRC4* genes) as well as mutations present in both, the metastatic and original surgical resection specimens (i.e., in *TP53*, *TAOK2*, and *MAP4K2* genes) [1]. Analyzing cancer genomes from seven rare types of metastatic adolescent and young adult cancers (including ONB) using whole exome sequencing [WES], whole-transcriptome sequencing, or OncoScan^™^, Cha et al. identified *TP53* missense mutation in a metastatic ONB sample, as well as a loss-of-function in *CDKN2C* gene [14]. Based on these results, they proposed CDK4/6 inhibitors, palbociclib and LY2835219, as potential treatment strategies [16,18]. Similarly, a recent comprehensive genomic study of Gay et al revealed alterations of *TP53*, *PIK3CA*, *NF1*, *CDKN2A*, and *CDKN2C* in ONBs [16]. The study of Wang et al. was first to report a case of recurrent ONB treated with a targeted therapy regimen determined after WES. Mutations in *EGFR*, *FGFR2*, *KDR*, and *RET* genes were detected, therefore the authors utilized a combination of cetuximab and sunitinib [15].

Considering the lack of standardized treatment guidelines, the potential advantages of targeted therapy approaches [8] and the paucity of data exploring the molecular pathogenesis of ONB, we explored potentially targetable biomarkers/pathways in a cohort of recurrent or metastatic ONBs, using multiplatform molecular profiling approach. We identified multiple protein biomarkers of response or resistance to classic chemotherapy and targeted therapy that could be useful in optimizing the cytotoxic chemotherapy and further improving personalized treatment of ONB.

## Materials and methods

### Patients and samples

This retrospective study included 23 formalin-fixed paraffin-embedded (FFPE) samples of the patients with recurrent or metastatic ONB (see S1 Excel File) profiled at the CLIA-certified laboratory, Caris Life Sciences (Phoenix, Arizona) in the period 2012–2017. Histologic diagnosis and review of results of immunohistochemical tests performed at the referring institutions to support the diagnosis of ONB were confirmed by a board certified pathologist (Z.G.) and appropriate slides were used for molecular profiling. Microdissection of tumor samples was performed when appropriate to enrich the tumor cell population.

Caris Life Sciences de-identified, remnant samples provided by participating investigators. Tumor profiling was performed and results were associated to a Subject ID. Because remnant tissue from previous samplings with no associated identifiers were utilized, this research was compliant with 45 CFR 46.101(b). Therefore, the project was deemed exempt from IRB oversight and consent requirements were waived.

### Immunohistochemistry (IHC)

Expression of predictive biomarkers was evaluated immunohistochemically using commercially available antibodies and detection kits by automated staining techniques (Benchmark XT, Ventana, Tucson, AZ): antibodies against androgen receptor (AR) [n = 18], topoisomerases 1 and 2 alpha (TOPO1, TOP2A) [n = 19], estrogen receptor (ER) [n = 18], progesterone receptor (PR) [n = 18], MET proto-oncogene, receptor tyrosine kinase (c-MET) [n = 13], human epidermal growth factor receptor 2 (HER2) [n = 19], tyrosine protein c-Kit receptor kinase (c-Kit) [n = 6], epidermal growth factor receptor (EGFR) [n = 5], phosphatase and tensin homolog (PTEN) [n = 18], O(6)-methylguanine methyltransferase (MGMT) [n = 19], P-glycoprotein (PGP) [n = 16], thymidylate synthase (TS) [n = 19], transducin-like enhancer of split 3 (TLE3) [n = 12], ribonucleotide reductase M1 (RRM1) [n = 15], serum protein acidic and rich in cysteine M (SPARC-M) [n = 13], tubulin beta-3 chain (TUBB3) [n = 14], anaplastic lymphoma kinase (ALK) [n = 4], breast cancer resistance protein (BCRP) [n = 4], excision repair cross-complementation group 1 protein (ERCC1) [n = 12], multidrug resistance associated protein 1 (MRP1) [n = 6], programmed cell death-1 (PD-1) [n = 8], platelet-derived growth factor receptor (PDGFR) [n = 4], and programmed death ligand-1 (PD-L1) [n = 10], tyrosine receptor kinase (pan-antiNTRK [TrkA+B+C]) [n = 6]. Scoring system and cutoffs for all antibodies were used as described in our previous studies [19,20] (S1 Table). All IHC assays were run along with both positive and negative controls.

### Copy number assays (fluorescence in situ hybridization [FISH] and chromogenic in situ hybridization [CISH])

FISH was used for evaluation of the *EGFR* status (Vysis LSI EGFR SpectrumOrange/CEP7 Spectrum Green Probe, Abbott) [n = 5] while *HER2* [n = 11] and *c-MET* [n = 9] genes were evaluated using CISH (dual EGFR DNP/CEP 7 DIG probes; INFORM HER2 Dual ISH DNA Probe Cocktail; commercially available c-MET and chromosome 7 DIG probe; Ventana, Tucson, AZ) as previously described [19,21]. The tumors were considered amplified for *HER2* when *HER2/CEP17* ratio >2 [22]; *EGFR* was amplified when *EGFR/CEP7* ratio >2 or >15 *EGFR* gene copies per cell were observed in >10% of analyzed cells [20]. *cMET* was amplified if >5 *cMET* copies on average were observed [19].

### DNA sequencing (Next-generation [NGS] and Sanger sequencing)

NGS was performed on genomic DNA isolated from 15 FFPE samples using the Illumina MiSeq platform (La Jolla, CA). The Illumina TruSeq Amplicon—Cancer Panel (TSACP) was used for amplifying specific genomic regions. The NGS panel covering 46 genes were tested on 10 ONB cases while five cases were explored using the extended NGS panel that covers 592 genes (available here: http://www.carismolecularintelligence.com/solid_tumors_international) [19,21].

For selected regions of v-Raf murine sarcoma viral oncogene homolog B (*BRAF*), V-Ki-ras2 Kirsten rat sarcoma viral oncogene homolog (*KRAS*), *c-KIT*, *EGFR*, and phosphatidylinositol 3-kinase catalytic subunit alpha (*PIK3CA*) genes Sanger sequencing was also used [19].

### Gene fusions

Six recent ONB cases were tested for gene fusions using Archer FusionPlex Solid Tumor Kit with Illumina MiSeq (Table 1, S2 Table, and online: http://www.carismolecularintelligence.com/solid_tumors_international).

**Table 1:** Results of *in situ* hybridization, sequencing and gene fusion assays.

Case	Age (sex)	*In situ* hybridization	Sequencing
**#1**	46 (M)	*EGFR* negative	*TP53H214Y*
**#2**	52 (F)	*HER2*, *cMET* negative	*c-KIT G565V; TP53 T155_V157del*
**#3**	71 (F)	*HER2*, *cMET* negative	*APC SNP A1474T*
**#4**	59 (F)	*HER2*, *cMET* negative	w.t.
**#5**	60 (M)	*HER2*, *cMET* negative	n/a
**#6**	53 (M)	n/a	w.t.
**#7**	63 (M)	n/a	*TP53c673-1G>T*
**#8**	51 (M)	*HER2*, *cMET* negative	*CTNBB1 S33_H36del;* [no gene fusion]
**#9**	52 (F)	n/a	w.t.
**#10**	68 (F)	*HER2*, *cMET* negative	w.t.
**#11**	43 (F)	*HER2*, *CMET* negative	*SMAD4 N468fs*
**#12**	73 (F)	n/a	*cMET L1321I (VUS); PDGFRA V546L (VUS)*
**#13**	29 (M)	n/a	n/a
**#14**	50 (F)	*EGFR* negative	n/a
**#15**	62 (M)	*HER2*, *cMET* negative	w.t.
**#16**	68 (F)	*EGFR* negative	n/a
**#17**	47 (M)	n/a	n/a
**#18**	84 (F)	*EGFR* negative	n/a
**#19**	47 (M)	*HER2* negative	*CTNNB1 S33P;* [no gene fusion]
**#20**	65 (F)	n/a	w.t.; [no gene fusion]
**#21**	68 (F)	n/a	*CDH1* D756Y (VUS), *FH* (K477dup); [no gene fusion]
**#22**	59 (F)	*EGFR* negative	*EGFR Q276R* (VUS); [no gene fusion]
**#23**	57 (M)	n/a	*EGFR T572R* (VUS); [gene fusion reaction failed]

VUS = Variant of unknown significance; w.t. = wild type; M = Male; F = Female; n/a = not available. EGFR: Epidermal growth factor receptor; HER2: Human epidermal growth factor receptor 2; cMET: MET proto-oncogene, receptor tyrosine kinase; APC: Adenomatous polyposis coli; TP53: Tumor suppressor p53.

### Whole genome RNA microarray

Whole-genome expression (RNA) was analyzed in four samples using Illumina cDNA-mediated annealing, selection, extension and ligation (DASL) process with the HumanHT-12 v4 beadChip (Illumina Inc., San Diego, CA) [21]. The RNA used for this analysis was extracted from FFPE samples by the Qiagen kit. The control used in this study consisted of three nerve RNAs from three healthy individuals pooled into a single sample. The over and under expression of transcripts in ONB samples were determined by taking the ratio of transcript in each ONB over the control. Transcripts were identified as significant if all the four ONB samples had consistently high or low ratio of expression when compared to control.

## Results

### Patients

The study included 23 patients (10 male and 13 female patients, age range: 29–84 years) with recurrent or metastatic ONB, profiled at Caris CLIA-certified laboratory in the period 2012–2017 (Table 1, S1–S3 Excel Files).

### Molecular profiling using IHC, ISH, sequencing and gene fusions

**Fig 1:**
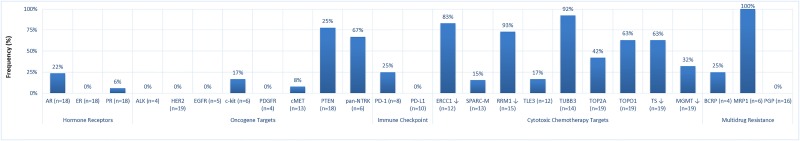
Profiling results of 25 biomarkers using immunohistochemistry (IHC) and *in situ* hybridization (ISH). AR: androgen receptor; ER: estrogen receptor; PR: progesterone receptor; ALK: anaplastic lymphoma kinase; HER2: Human epidermal growth factor receptor; EGFR: epidermal growth factor receptor; c-Kit: tyrosine protein c-Kit receptor kinase; PDGFR: platelet-derived growth factor receptor; c-MET: MET proto-oncogene, receptor tyrosine kinase; PTEN: phosphatase and tensin homolog; pan-NTRK: tyrosine receptor kinase; PD-1: programmed cell death-1; PD-L1: programmed death ligand-1; ERCC1: excision repair cross-complementation group 1 protein; SPARC-M: Serum protein acidic and rich in cysteine M; RRM1: ribonucleotide reductase M1; TLE3: transducin-like enhancer of split 3; TUBB3: tubulin beta-3 chain; TOPO1 and TOP2A: topoisomerases 1 and 2 alpha; TS: thymidylate synthase; MGMT: O(6)-methylguanine methyltransferase; BCRP: breast cancer resistance protein; MRP1: multidrug resistance associated protein 1; PGP: P-glycoprotein.

The IHC, ISH, and sequencing results are summarized in Fig 1, Table 1 and S2 Excel File.

**Fig 2:**
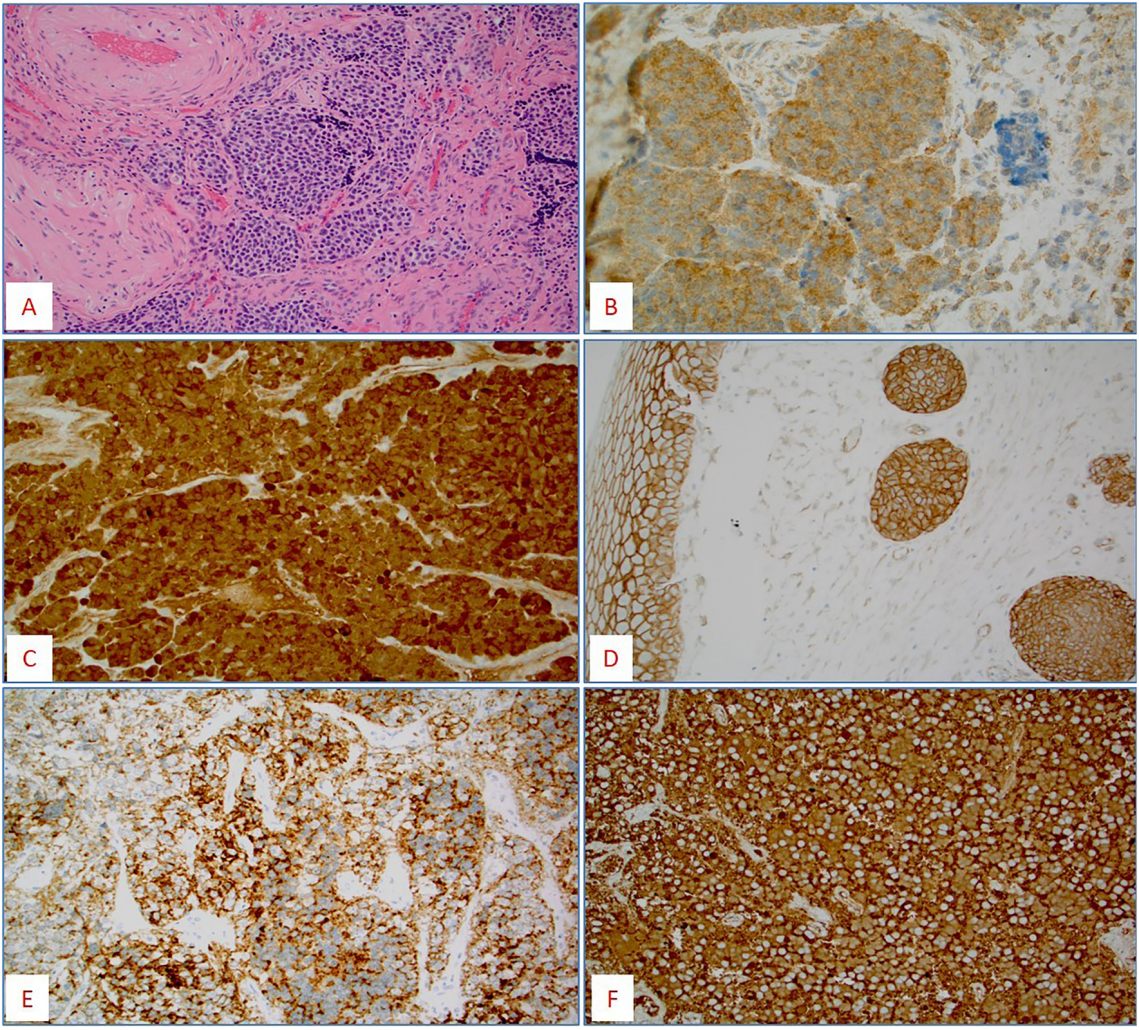
**(A)**: Hematoxylin and Eosin (H&E) figure of a case with upregulation of *CD24* gene by microarray, confirmed by **CD24 protein** overexpression in the tumor cells **(B)**; **(C)** A case of olfactory neuroblastoma (ONB) with *CTNNB1* mutation [S33_ H36del] confirmed by the nuclear expression of **β-catenin**; Another case of ONB with *CTNNB1* mutation [S33P] with retained cytoplasmic/membranous expression of **β-catenin protein (D)**; A case of recurrent ONB with **pNTRK** overexpression **(E)** and overexpression of **TUBB3 (F)**.

No cases expressed PD-L1 (0/10). Multiple protein biomarkers of response or resistance to classic chemotherapy drugs were identified: PD-1 positive tumor infiltrating lymphocytes in 25% (2/8), low ERCC1 (cisplatin sensitivity [23] in 83% (10/12), high TOPO1 (irinotecan sensitivity [24] in 63% [12/19], high TUBB3 (vincristine resistance [25] in 93% [13/14] (Fig 2F), and high MRP1 (multidrug resistance) in 100% (6/6). Four out of six tested ONBs (67%) were positive for pNTRK (Fig 2E).

Mutations (pathogenic and variants of unknown significance [VUS]) were detected in 10/16 (63%) ONBs including tumor suppressor p53 (*TP53*) [3 cases], beta-catenin 1 gene (*CTNNB1*), *EGFR* [2 cases, respectively], while single cases harbored Adenomatous Polyposis Coli (*APC*), *cKIT*, *cMET*, Platelet Derived Growth Factor Receptor Alpha (*PDGFRA*), *CDH1 (E-cadherin*), Fumarate Hydratase *(FH)* and *SMAD4* gene mutations (Table 1). *CTNNB1* gene alterations were further evaluated by the IHC (Fig 2C and 2D).

None of the cases harbored gene amplifications of *EGFR*, *HER2* and *cMET* genes. Also, gene fusions were not identified in any of the 6 successfully tested ONBs (the panel of fusion genes is available in S2 Table).

### Microarray results

When compared with the control tissue, 21 genes were consistently over-expressed and 19 genes consistently downregulated by an average of 10 fold. Some of the upregulated genes, such as Secretogranin II (*SCG2*), stem cell marker cluster of differentiation 24 [*CD24*] (Fig 2A), and insulin-like growth factor binding protein 2 (*IGFBP-2*), and downregulated genes (ATP-binding cassette transporter 8 [*ABCA8*] have been described to play a role in different malignancies, and were not hitherto described in ONBs (Table 2). Among the downregulated genes, *GHR* is a novel observation as most studies associate *GHR* over-expression as a risk factor for cancer. *CD24* gene expression has been confirmed by the IHC (Fig 2B).

**Table 2:** Selected genes’ mRNA expression detected in olfactory neuroblastoma samples using Illumina array.

Gene	Location	Name/Function	Relative expression ratio*
*SCG2*	2q36.1	Secretogranin II/chromogranin/secretogranin family of neuroendocrine secretory proteins	5.6–6.7
*CD24*	6q21	Modulates growth and differentiation of hematopoietic cells	5.5–7.3
*IGFBP-2*	2q35	Insulin-Like Growth Factor Binding Protein 2/promotes cell growth	3.9–7.7
*ABCA8*	17q24.2	Transports various molecules across extra- and intracellular membranes	0.18–0.26
*GHR*	5p13.1	Growth hormone receptor	0.28–0.29

* Compared with normal neural tissue.

Abbreviations: SCG2: Secretogranin II (member of neuroendocrine secretory proteins; the full-length protein is cleaved to produce the active peptide secretoneurin); CD24 (hematopoietic and stem cell marker); IGFBP-2: Insulin-like growth factor binding protein 2 (an oncogene in most human epithelium cancers); ABCA8 (ATP-binding cassette transporter 8; GHR: Growth hormone gene.

In order to better understand the pathways perturbed in ONB, we extended the list of over- and under-expressed genes to those that were consistently over- and under-expressed by two fold followed by functional classification of these genes using the Panther website (http://pantherdb.org) and the Reactome database (see S3 Excel File for the list of genes). Overall, 183 genes were found to be downregulated and 146 were over-expressed. At the nominal p-value of 0.05, fifty-nine Reactome pathways were shown to be enriched with the 183 downregulated genes in ONB including 10 genes in the organization of extra cellular matrix and 4 genes in the cell junction organization. Pathway analysis of the 146 upregulated genes identified 100 enriched pathways including 13 genes in the Cell Cycle pathway, 10 genes in the *TP53* pathway, 8 genes in Chromatin Modifying Enzyme pathway (The list of all enriched pathways can be found in S3 Excel File).

## Discussion

Recent studies demonstrated potential therapeutic benefits of comprehensive molecular profiling for the patients with advanced and/or metastatic cancers [16,26–28]. In this study, we explored a wide range of potentially targetable biomarkers/pathways in recurrent or metastatic ONB samples using multiple molecular profiling platforms, including IHC, ISH, expression microarray and NGS. The sequencing results showed mutations in *TP53* (n = 3/16), *CTNNB1* (n = 2/16), *EGFR* (n = 2/16), *APC*, *cKIT*, *cMET*, *PDGFRA*, *CDH1*, *FH*, and *SMAD4* genes (n = 1/16, respectively). Multiple genes within the Wnt/β-catenin signaling pathway including *CTNNB1*, *APC* and *CDH1* exhibited mutations within this cohort. Loss-of-function mutations in these genes lead to deregulated Wnt/β-catenin signaling and excessive stem cell renewal/proliferation, and are associated with metastatic disease [29,30]. However, we found no targetable Wnt pathway enrichment in our cohort using whole-genome expression assay. The potential of several anti-cancer drugs has been explored by targeting different stages or components of Wnt/β-catenin signaling pathway with limited success [31,32]. The role of *cKIT* and *PDGFRA* mutations has been, most notably, investigated in gastrointestinal stromal tumors (GIST), where these two mutations are mutually exclusive [33]. Imatinib (tyrosine-kinase inhibitor) response in GIST patients depends not only on the protein expression, but also on the type of mutation in *KIT* and *PDGFRA* genes [33,34]. One ONB case in our study had a pathogenic *cKIT* mutation while another harbored VUS *PDGFRA* mutation. Both *EGFR* gene mutations in our study were VUS while *EGFR* amplification was not observed in any of the tested cases. These results indicate a limited therapeutic benefit of EGFR inhibitors in ONB patients. Mutations in *TP53* gene were also detected in other studies that performed DNA sequence analysis in ONB samples [13–17]. Due to the disease progression in those patients, a role of *TP53* mutation as an unfavorable prognostic and predictive factor in ONB has been suggested [18]. Of note, tumors harboring *TP53* mutations may be sensitive to WEE kinase inhibitors acting against G2-M checkpoint regulators of the cell cycle WEE1 and CHK1 [35].

In addition, our microarray analysis revealed up or downregulation of several genes previously implied in carcinogenesis but not previously described in ONBs, including *CD24*, *SCG2*, and *IGFBP-2* among the upregulated genes, and *ABCA8* and *GHR* among the downregulated genes. In a recent study by Dvorak et al. a comprehensive expression analysis of all members of the ABC transporter genes across multiple cancers showed that *ABCA8* downregulation was more observed in higher grade and its upregulation was associated with lower grade tumors [36]. This is consistent with the data presented in our paper that *ABCA8* was consistently downregulated in all four high-grade ONB samples. Interestingly, the study by Dvorak et al. was able to show that out of all 49 ABC transporters that were investigated in various tumors, *ABCA8* and four others (*ABCC7*, *ABCC8*, *ABCA3*, and *ABCA12*) were among the most dysregulated *ABC* genes [36]. *ABCA8* has also been studied by others in relation to cancer and it has been found to be downregulated in multidrug resistant ovarian cancer cell lines [37] as well as in breast and prostate cancer [38,39]. In one study, *ABCA8* was found to be upregulated in a subtype of medulloblastoma defined as Sonic hedgehog (SHH) and downregulated in the subtype defined as Wnt signaling [40]. It should be noted that the downregulation of *GHR* gene in ONB was an unexpected result, as most studies in the literature associate *GHR* upregulation with increased cancer risk [41].

More recently, immune checkpoint inhibitors (anti-PD-1/PD-L1) have revolutionized the treatment of many tumors with most remarkable benefits in the patients with melanoma, non-small cell lung carcinoma, renal cell carcinoma, bladder carcinoma, and classical Hodgkin lymphoma [42]. Our study is the first to report on the lack of PD-L1 expression in ONB samples, which makes these patients less likely to respond to anti-PD-1/PD-L1 drugs.

Recently, Gay et al. comprehensively profiled 41 samples of ONBs identifying potential targets in the mTOR, CDK and growth factor signaling pathways [16]. Other, small studies on molecular characteristics of ONB showed variable and largely inconsistent results. In the study of Weiss et al. 119 somatically lost genes and 45 gained or amplified genes were reported in a metastatic ONB sample using whole genome sequencing [13]. Seven somatic short nucleotide variants (SNVs) were validated by Sanger sequencing. Specific mutations in *KDR*, *MYC*, *SIN3B*, and *NLRC4* genes were present only in the metastatic ONB sample, while mutations in *TP53*, *TAOK2* and *MAP4K2* genes were present in both the metastatic and original surgical resection specimens [13]. Our study confirmed some of these mutations (e.g. *TP53*) but failed other mutations including *MYC* mutation. In contrast to other genetic alterations (copy number variations, chromosomal translocations, increased enhancer activity), *MYC* gene mutations are uncommon [43], but have been described in some cancers (e.g. lymphomas) [44]. Furthermore, the described *MYC* mutation in ONB [13] has not been verified in the COSMIC database (Catalog of Somatic Mutations in Cancer). Notably, none of our ONB cases harbored *MYCN* gene amplification, a hallmark of pediatric neuroblastomas [45]. Further studies should definitely elucidate the role of *MYC* gene(s) in ONBs.

Using two different genomics platforms, Cha et al. reported a *TP53* missense mutation in a metastatic ONB sample and a loss-of-function in *CDKN2C* gene [14]. Wang et al. detected mutations in *EGFR*, *FGFR2*, *KDR*, and *RET* genes in a recurrent ONB sample, using WES. In addition, *EGFR* and *KDR* genes were over-expressed in the tumor tissue [15].

Despite over-expression of the tropomyosin receptor kinase receptor family (NTRK) observed in 4/6 tested ONB cases, we did not detect any fusion of either one of the three *NTRK* genes (*NTRK1*, *NTRK2*, and *NTRK3*). Cancers with overexpression of *NTRK* driven by gene fusions had been successfully treated with novel NTRK kinase inhibitors [46] but it remains unclear if a “constitutive” overexpression such as this observed in ONB would offer any treatment advantages. Interestingly, one of the cases with NTRK overexpression exhibited TUBB3 positivity and was CD24 positive (Fig 2) indicating a potential therapeutic benefit of retinoid-based therapy [47].

*ALK* (anaplastic lymphoma receptor tyrosine kinase) gene alterations have been reported in various cancers including pediatric neuroblastomas [48]. We found no *ALK* gene alterations (mutations or fusions) or over-expression of Alk protein in any of the tested ONB cases, so these patients are unlikely to benefit from the ALK inhibitors.

In several studies, alternative approaches to ONB treatment, especially in metastatic or recurrent cases, have been explored. A combination of cetuximab (a monoclonal antibody to EGFR) and sunitinib (small-molecule inhibitor of receptor tyrosine kinases [RTKs] including kinase insert domain receptor [KDR], fibroblast growth factor receptor 2 [FGFR2], and RET RTK) was selected as a treatment regimen in a case of recurrent ONB, after WES analysis. One month after this treatment, a complete response was observed in the patient [15]. In another study, a significant improvement of clinical symptoms and disease stabilization for 15 months were observed after treatment with sunitinib in a patient with progressive ONB [49]. Furthermore, imatinib mesylate was reported as a potential second-line treatment for inducing long-term remission in heavily pretreated ONB patients [50]. Young et al. went a step further and explored the efficacy of several combinations of targeted drugs in human ONB cell line TC268 [51]. The combinations of AEW541 (insulin-like growth factor 1 [IGF-1] inhibitor) and FS114 (ribosomal protein S6 kinase beta-1 [S6K1] inhibitor) or sunitinib and FS115 (S6K1 inhibitor) were the most effective according to their results [51]. In addition, Sabongi et al. described a case with multiple recurrences of ONB adjuvantly treated with the radiolabeled-somatostatin analogue, ^177^Lu-DOTA-TATE. After three cycles of ^177^Lu-DOTA-TATE treatment, the stabilization of the disease was reported [52]. Finally, Mao et al. investigated the role of SHH signaling pathway in the development of ONB, by treating ONB cell lines (TC-268 and JFEN) with cyclopamine, a selective inhibitor of the SHH pathway [53]. After the cyclopamine treatment, inhibited ONB cell proliferation and colony formation, induced ONB cell cycle arrest and apoptosis, downregulated expression of SHH signaling components, i.e. *PTCH1* and *Gli1*, and *CCND1* (cyclin D1, cycle-related regulator), as well as upregulated *p21* expression were observed *in vitro* [53].

The efficacy of classic chemotherapy in ONBs remains unclear [2,4]. Chemotherapy alone, or in combination with radiotherapy, is often limited to advanced and surgically inoperable ONB cases [54]. Our IHC results indicate that several biomarkers may be used in tailoring the classical cytotoxic drugs including cisplatin and irinotecan sensitivity and vincristine resistance.

Our study have several limitations including a small sample size and lack of clinical (follow-up) data. In addition, all samples were not tested with all methodologies as these have been dynamically changing per molecular testing advances/improvements. This may result in insufficient and biased therapeutic implications.

Although our data indicate limited therapeutic options in patients with advanced and/or metastatic ONBs, several potential biomarkers that could tailor both targeted (e.g., Wnt and cKIT/PDGFRA) and classical therapeutic options merit further research. The therapeutic benefits of immune checkpoint inhibitors are less likely due to the low or lack of PD-1/PD-L1 expression.

## Supporting information

S1 Table The list of antibodies used for immunohistochemical biomarkers profiling. (DOCX)

S2 Table Gene fusions tested in six olfactory neuroblastoma samples using Archer FusionPlex Solid Tumor Kit with Illumina MiSeq. (DOCX)

S1 Excel File Olfactory neuroblastoma database. (XLSX)

S2 Excel File Biomarkers profiled using immunohistochemistry and in-situ hybridization assays. (XLSX)

S3 Excel File Microarray data. (XLSX)
