# Supplementary material for: Comprehensive molecular profiling of advanced/metastatic olfactory neuroblastomas
Source: PLoS One. 2018 Jan 11;13(1):e0191244. doi: 10.1371/journal.pone.0191244 (PMC5764485; doi:10.1371/journal.pone.0191244)
Supplement: S1 Table — (DOCX) [file pone.0191244.s001.docx]

**S1 Table.** **The list of antibodies used for immunohistochemical biomarkers profiling.**

| **Biomarker** | **Manufacturer** | **Localization** | **Threshold** |
| --- | --- | --- | --- |
| Androgen receptor (AR) | Leica Biosystems | Nuclear | = 0+ or <10% or ≥1+ and ≥10% |
| Estrogen receptor (ER) | Ventana | Nuclear | = 0+ or = 0% or ≥1+ and ≥1% |
| Progesterone receptor (PR) | Ventana | Nuclear | = 0+ or = 0% or ≥1+ and ≥1% |
| Epidermal growth factor receptor (EGFR) | Dako | Membrane | = 0+ or =1+ and =10% or  = 2+ and =10% |
| Human epidermal growth factor receptor 2 (HER2) | Ventana | Membrane | <1+ or = 2+ and ≤10% or ≥3+  and >10% |
| MET proto-oncogene, receptor tyrosine kinase (c-MET) | Ventana | Membrane | <50% or <2+ or ≥2+ and ≥50% |
| O(6)-methylguanine methyltransferase (MGMT) | Invitrogen | Nuclear | =0+ or ≤35% or ≥1+ and >35% |
| P-glycoprotein (PGP) | Invitrogen | Membrane | =0+ or <10% or ≥1+ and ≥10% |
| Phosphatase and tensin homolog (PTEN) | Dako | Nuclear, membrane, cytoplasm | =0+ or ≤50% or ≥1+ and >50% |
| Ribonucleotide reductase M1 (RRM1) | Protein Tech | Cytoplasm | =0+ or <50% or <2+ or ≥2+ and ≥50% |
| Serum protein acidic and rich in cysteine M and P (SPARC-M and P) | Monoclonal, R&D Systems; Polyclonal, Exalpha | Cytoplasm | <30% or <2+ or ≥2+ and ≥30% |
| Transducin-like enhancer of split 3 (TLE3) | Santa Cruz | Nuclear | <30% or <2+ or ≥2+ and ≥30% |
| Topoisomerase 2 alpha (TOPO2A) | Leica Biosystems | Nuclear | =0+ or <10% or ≥1+ and ≥10% |
| Topoisomerase 1 (TOPO1) | Leica Biosystems | Nuclear | =0+ or <30% or <2+ or ≥2+ and ≥30% |
| Thymidylate synthase (TS) | Invitrogen | Nuclear, cytoplasm | =0+ or ≤3+ and <10% or ≥1+ and ≥10% |
| Tubulin beta-3 chain (TUBB3) | Covance | Cytoplasm | <30% or <2+ or ≥2+ and ≥30% |
| Excision repair cross-complementation group 1 protein (ERCC1) | Abcam | Nuclear | <2+ or ≤3+ and <10% or = 2+ and <50% or ≥3+ and ≥10% or ≥2+ and ≥50% |
| Tyrosine protein c-Kit receptor kinase (c-Kit) | Dako | Membrane | 10%, 1+ |
| Anaplastic lymphoma kinase (ALK) | Ventana | membrane | <3+ or ≥3+ and ≥1% |
| Breast cancer resistance protein (BCRP) | Santa Cruz | Membrane | =0+ or =1+ and <10% or ≥1+ and ≥10% |
| Multidrug resistance associated protein 1 (MRP1) | Novocastra | membrane | =0+ or <10% or ≥1+ and ≥10% |
| Programmed cell death-1 (PD-1) | Cell Marque | Membrane/cytoplasm | =0+ or ≥1+ |
| Platelet-derived growth factor receptor (PDGFRA) | ThermoFisher Scientific | membrane | =0+ and =100% or ≥2+ and ≥30% |
| Programmed death ligand-1 (PD-L1) | Ventana | Membrane/cytoplasm | ≥5% 2+ |
| Tyrosine receptor kinase (TrkA+B+C) | Abcam | membrane | =0+ or =100+ or ≥1+ and ≥1% |
