# Supplementary material for: Comprehensive molecular profiling of advanced/metastatic olfactory neuroblastomas
Source: PLoS One. 2018 Jan 11;13(1):e0191244. doi: 10.1371/journal.pone.0191244 (PMC5764485; doi:10.1371/journal.pone.0191244)
Supplement: S2 Table — (DOCX) [file pone.0191244.s002.docx]

**S2 Table.** **Gene fusions tested in six olfactory neuroblastoma samples using Archer FusionPlex Solid Tumor Kit with Illumina MiSeq.**

| **Gene** | **Transcript** | **Exons** | **Direction** | **Type** |
| --- | --- | --- | --- | --- |
| *AKT3* | NM_005465 | 1, 2, 3 | 5' | Fusion |
| *ALK* | NM_004304 | 19, (intron19), 20, 21, 22 | 5' | Fusion |
| *ARHGAP26* | NM_015071 | 2, 10, 11, 12 | 5' | Fusion |
| *AXL* | NM_021913 | 19, 20 | 3' | Fusion |
| *BRAF* | NM_004333 | 7, 8 | 3' | Fusion |
| *BRAF* | NM_004333 | 7, 8, 9, 10, 11, 12 | 5' | Fusion |
| *BRAF* | NM_004333 | 15 | 5' | Fusion |
| *BRD3* | NM_007371 | 9, 10, 11, 12 | 3' | Fusion |
| *BRD4* | NM_014299 | 10, 11 | 3' | Fusion |
| *EGFR* | NM_005228 | 7, 9, 16, 20 | 5' | Fusion |
| EGFR | NM_005228 | 24, 25 | 3' | Fusion |
| *ERG* | NM_004449 | 2, 3, 4, 5, 6, 7, 8, 9, 10, 11 | 5' | Fusion |
| *ESR1* | NM_001122742 | 3, 4, 5, 6 | 3' | Fusion |
| *ETV1* | NM_004956 | 3, 4, 5, 6, 7, 8, 9, 10, 11, 12, 13 | 5' | Fusion |
| *ETV4* | NM_001986 | 2, 4, 5, 6, 7, 8, 9, 10 | 5' | Fusion |
| *ETV5* | NM_004454 | 2, 3, 7, 8, 9 | 5' | Fusion |
| *ETV6* | NM_001987 | 1, 2, 3, 4, 5, 6 | 3' | Fusion |
| *ETV6* | NM_001987 | 2, 3, 5, 6, 7 | 5' | Fusion |
| *EWSR1* | NM_005243 | 4, 5, 6, 7, 8, 9, 10, 11, 12, 13, 14 | 3' | Fusion |
| *FGFR1* | NM_015850 | 2, 8, 9, 10, 17 | 5' | Fusion |
| *FGFR2* | NM_000141 | 2, 8, 9, 10 | 5' | Fusion |
| *FGFR2* | NM_000141 | 17 | 3' | Fusion |
| *FGFR3* | NM_000142 | 17, Intron 17 | 3' | Fusion |
| *FGFR3* | NM_000142 | 8, 9, 10 | 5' | Fusion |
| *FGR* | NM_005248 | 2 | 5' | Fusion |
| *INSR* | NM_000208 | 20, 21, 22 | 3' | Fusion |
| *INSR* | NM_000208 | 12, 13, 14, 15, 16, 17, 18, 19 | 5' | Fusion |
| *MAML2* | NM_032427 | 2, 3 | 5' | Fusion |
| *MAST1* | NM_014975 | 7, 8, 9, 18, 19, 20, 21 | 5' | Fusion |
| *MAST2* | NM_015112 | 2, 3, 5, 6 | 5' | Fusion |
| *MET* | NM_000245 | 13 | 3' | Fusion |
| *MSMB* | NM_002443 | 2, 3, 4 | 3' | Fusion |
| *MUSK* | NM_005592 | 7, 8, 9, 11, 12, 13, 14 | 5' | Fusion |
| *MYB* | NM_001130173 | 7, 8, 9, 11, 12, 13, 14, 15, 16 | 3' | Fusion |
| *NOTCH1* | NM_017617 | 2, 4, 29, 30, 31 | 3' | Fusion |
| *NOTCH1* | NM_017617 | 26, 27, 28, 29 (internal exon 3-27 deletion) | 5' | Fusion |
| *NOTCH2* | NM_024408 | 5, 6, 7 | 3' | Fusion |
| *NOTCH2* | NM_024408 | 26, 27, 28 | 5' | Fusion |
| *NRG1* | NM_004495 | 1, 2, 3, 6 | 5' | Fusion |
| *NTRK1* | NM_002529 | 8, 10, 11, 12, 13 | 5' | Fusion |
| *NTRK2* | NM_006180 | 11, 12, 13, 14, 15, 16, 17 | 5' | Fusion |
| *NTRK3* | NM_002530 | 13, 14, 15, 16 | 5' | Fusion |
| *NTRK3* | NM_001007156 | 15 | 5' | Fusion |
| *NUMBL* | NM_004756 | 3 | 5' | Fusion |
| *NUTM1* | NM_175741 | 3 | 5' | Fusion |
| *PDGFRA* | NM_006206 | 10, 11, 12, 13, 14, | 5' | Fusion |
| *PDGFRB* | NM_002609 | 8, 9, 10, 11, 12, 13, 14 | 5' | Fusion |
| *PIK3CA* | NM_006218 | 2 | 5' | Fusion |
| *PKN1* | NM_002741 | 10, 11, 12, 13 | 5' | Fusion |
| *PPARG* | NM_015869 | 1, 2, 3 | 5' | Fusion |
| *PRKCA* | NM_002737 | 4, 5, 6 | 5' | Fusion |
| *PRKCB* | NM_002738 | 3 | 5' | Fusion |
| *RAF1* | NM_002880 | 4, 5, 6, 7, 9 | 3' | Fusion |
| *RAF1* | NM_002880 | 4, 5, 6, 7, 9, 10, 11, 12 | 5' | Fusion |
| *RELA* | NM_021975 | 3, 4 | 5' | Fusion |
| *RET* | NM_020630 | 8, 9, 10, 11, 12, 13 | 5' | Fusion |
| *ROS1* | NM_002944 | 31, 32, 33, 34, 35, 36, 37 | 5' | Fusion |
| *RSPO2* | NM_178565 | 1, 2 | 5' | Fusion |
| *RSPO3* | NM_032784 | 2 | 5' | Fusion |
| *TERT* | NM_198253 | 2 | 5' | Fusion |
| *TFE3* | NM_006521 | 2, 3, 4, 5, 6 | 3' | Fusion |
| *TFE3* | NM_006521 | 2, 3, 4, 5, 6, 7, 8 | 5' | Fusion |
| *TFEB* | NM_007162 | 1, 2 | 5' | Fusion |
| *THADA* | NM_022065 | 28 | 3' | Fusion |
| *TMPRSS2* | NM_005656 | 1, 2, 3, 4, 5, 6 | 3' | Fusion |
| *TMPRSS2* | NM_001135099 | 1 | 3' | Fusion |
